# Probing Chemical Complexity of Amyloid Plaques in Alzheimer’s Disease Mice using Hyperspectral Raman Imaging

**DOI:** 10.1021/acschemneuro.3c00607

**Published:** 2023-12-14

**Authors:** Dušan Mrđenović, Benjamin F. Combes, Ruiqing Ni, Renato Zenobi, Naresh Kumar

**Affiliations:** † Department of Chemistry and Applied Biosciences, ETH Zürich, Vladimir-Prelog-Weg 1−5/10, 8093 Zürich, Switzerland; § Institute for Regenerative Medicine, University of Zürich, Wagistrasse 12, 8952 Schlieren, Switzerland; # Institute for Biomedical Engineering, University of Zurich and ETH Zurich, Wolfgang-Pauli-Strasse 27, 8093 Zürich, Switzerland

**Keywords:** Amyloid plaque, label-free imaging, sub-μm scale, hyperspectral Raman microscopy, Alzheimer’s disease, transgenic mouse model

## Abstract

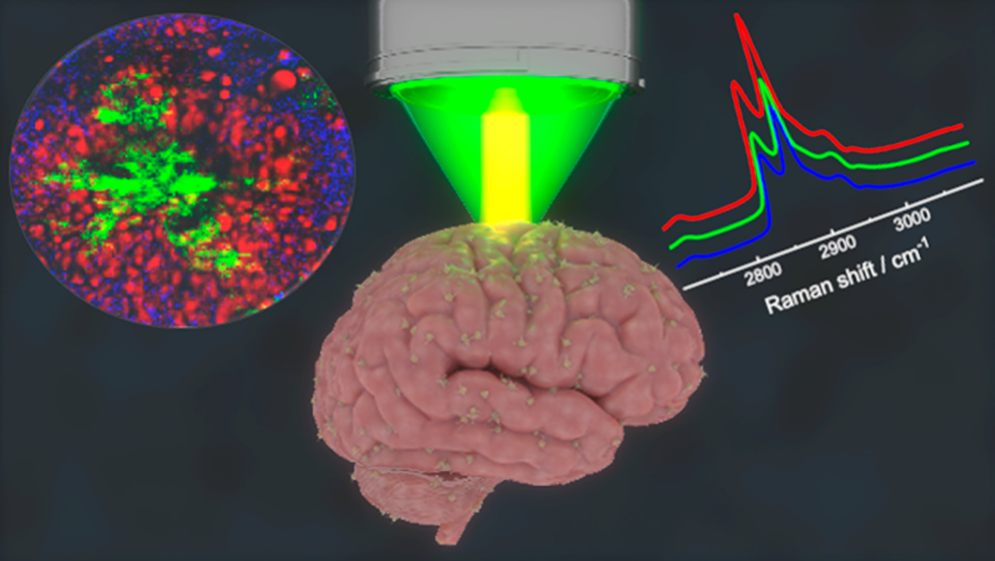

One of the distinctive pathological features of Alzheimer’s disease (AD) is the deposition of amyloid plaques within the brain of affected individuals. These plaques have traditionally been investigated using labeling techniques such as immunohistochemical imaging. However, the use of labeling can disrupt the structural integrity of the molecules being analyzed. Hence, it is imperative to employ label-free imaging methods for noninvasive examination of amyloid deposits in their native form, thereby providing more relevant information pertaining to AD. This study presents compelling evidence that label-free and nondestructive confocal Raman imaging is a highly effective approach for the identification and chemical characterization of amyloid plaques within cortical regions of an arcAβ mouse model of AD. Furthermore, this investigation elucidates how the spatial correlation of Raman signals can be exploited to identify robust Raman marker bands and discern proteins and lipids from amyloid plaques. Finally, this study uncovers the existence of distinct types of amyloid plaques in the arcAβ mouse brain, exhibiting significant disparities in terms of not only shape and size but also molecular composition.

## 1 Introduction

Alzheimer’s disease (AD) is a neurodegenerative disorder characterized by cognitive impairments, including memory loss, communication difficulties, and personality changes.^1^ It is the most prevalent form of dementia, which is estimated to globally impact over 152 million individuals by 2050.^2^ Consequently, addressing the urgent challenge of developing effective diagnostic and therapeutic interventions for AD is crucial to public health. An important pathological feature of AD is the abnormal accumulation of amyloid β deposits, also known as plaques, in the brain.^3^ Diagnostic biomarkers for AD have traditionally targeted amyloid β and tau proteins in brain,^4,5^ cerebrospinal fluid,^6,7^ and blood,^8,9^ utilizing labeling techniques that involve the attachment of antibodies or fluorophores to these proteins. However, the use of labeling molecules can alter the structure of the analyte, making accurate diagnosis difficult. Currently, the gold standard for AD analysis involves post-mortem examination of brain tissue through histology and immunohistochemical staining.^10,11^ However, this method is labor-intensive and time-consuming and carries the risk of sample dehydration and deformation, necessitating the exploration of alternative approaches.^12^

In response to these challenges, label-free spectroscopic techniques have emerged as promising methods for imaging amyloid plaques. These include confocal optical spectroscopy,^13^ micro-Fourier transform infrared spectroscopy (μFTIR),^14^ mid-infrared photothermal microscopy,^15^ coherent anti-Stokes Raman spectroscopy (CARS),^16^ stimulated Raman scattering spectroscopy (SRS),^12,17^ and tip-enhanced Raman spectroscopy (TERS).^18−22^ While infrared (IR) spectroscopy generally provides stronger signals than Raman spectroscopy, it is limited by strong absorption of IR light by water in the physiological environment. On the other hand, Raman signals in biological samples are typically weak, requiring long acquisition times to achieve a satisfactory signal-to-noise ratio, which extends the measurement span in spontaneous Raman microscopy. CARS and SRS address this limitation by exploiting the coherent nature of nonlinear optical processes, enhancing the particular biomolecular Raman signals by several orders of magnitude.^17^ However, compared to confocal Raman microscopy, CARS and SRS require expensive equipment, have poor spectral resolution, and may induce sample photodamage due to high peak-intensity excitation from pulsed lasers.^23^

Achieving spatially resolved chemical analysis through Raman imaging relies on the identification of reliable Raman marker bands specific to the target molecule. This task becomes challenging when dealing with complex biological specimens, such as amyloid plaques in brain tissues. For instance, in lipid samples, the intensity ratio of Raman bands at 1440 and 1650 cm^–1^ is commonly used to assess lipid unsaturation.^24,25^ However, this approach lacks reliability when analyzing samples that contain both lipids and proteins because the amide II and amide I bands of proteins overlap with the lipid signals at 1440 and 1650 cm^–1^, respectively. Similarly, the spectral region between 2860 and 2980 cm^–1^ has been employed as a Raman marker band for proteins in some studies,^26^ despite the presence of lipid signals in the same region.^27^ Therefore, to avoid misinterpretation of the data, it is crucial to utilize Raman marker bands that are uniquely associated with the vibrations of target analytes.

In this study, we demonstrate label-free and nondestructive hyperspectral confocal Raman imaging of amyloid plaques in the brains of 18-month-old transgenic arcAβ mice, a model for AD,^28^ with sub-μm resolution. While previous reports proposed using μFTIR imaging to locate amyloid plaques over a large sample area before zooming in with a Raman microscope,^14^ our findings demonstrate the direct identification of amyloid plaques using confocal Raman imaging alone, without the need for any complementary techniques. Importantly, our approach utilizes a standard confocal Raman microscope readily available in many laboratories worldwide, eliminating the requirement for prohibitively expensive SRS or CARS equipment. Furthermore, we show that the spatial correlation between multiple Raman marker bands enables reliable differentiation of amyloid plaques from non-amyloid proteins and lipids. Our results reveal significant differences in the content of proteins and lipids within amyloid plaques, suggesting the existence of distinct molecular compositions among different types of plaques. These novel insights, revealed through hyperspectral Raman imaging, broaden our understanding of the chemical complexity of amyloid plaques in the brains of arcAβ mouse models of AD.

## 2 Results and Discussion

**Figure 1:**
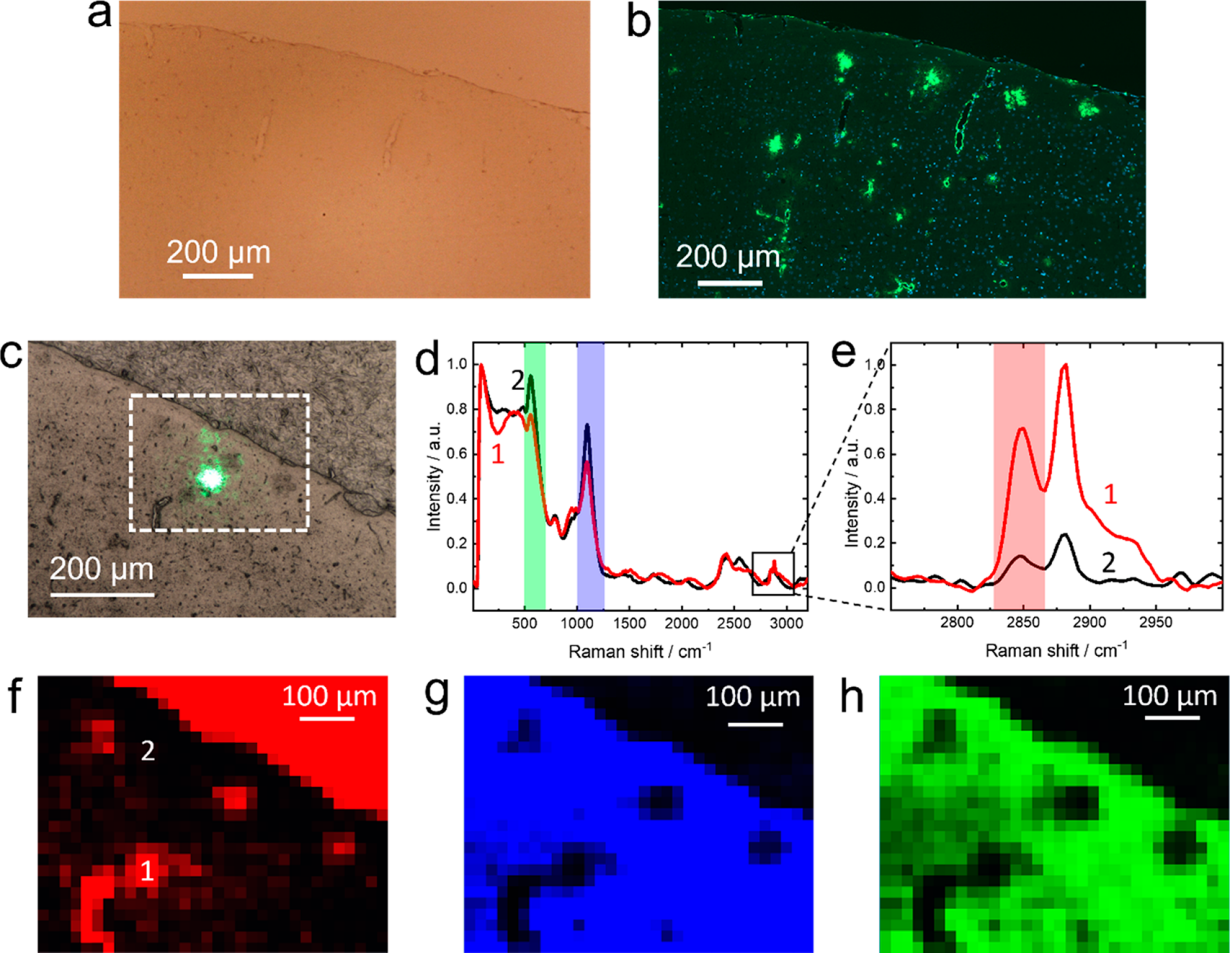
(a) Optical microscopy and (b) fluorescence microscopy images of a 1.5 μm-thick arcAβ mouse brain slice (cortex). The fluorescence image confirms the presence of amyloid plaques in the cortex of arcAβ mouse. (c) Optical image of the cortex region of an arcAβ mouse brain slice adjacent to the one shown in panel a. Focal spot of the green excitation laser is also visible in the image. (d, e) Raman spectra recorded at the position of an amyloid plaque (1) and away (2) from it (marked in panel f). Laser power: 77 mW. Acquisition time: 1 s. Step size: 20 μm. Confocal Raman images of the amyloid plaques in the cortex of the arcAβ mouse brain slice constructed using the intensity of (f) 2848, (g) 1097, and (h) 557 cm^–1^ Raman bands highlighted in red, blue, and green in panels d and e.

An optical micrograph of a 1.5 μm-thick sagittally cut arcAβ mouse brain slice placed on a glass coverslip is shown in Figure 1a. The presence of amyloid plaques (labeled with 6E10 antibodies and Alexa488) in the arcAβ mouse brain was confirmed using an Axio Observer Z1 fluorescence microscope ( 20× magnification, Figure 1b). For label-free visualization of amyloid plaques in the mouse brain using confocal Raman microscopy, reliable Raman marker bands need to be identified first. Since several amyloid plaques in the arcAβ mouse brain slice, shown in Figure 1a, are >10 μm in size (Figure 1b) and the slice itself is only 1.5 μm-thick, the same plaques are also expected to be present in the slice adjacent to it. An unlabeled mouse brain slice cut adjacent to the one in Figure 1a is shown in Figure 1c. The fluorescence image shown in Figure 1b was used as a guide to locate the amyloid plaques for Raman analysis in the unlabeled brain slice. To measure a large sample area (marked in Figure 1c), Raman imaging was performed with an objective lens of 5× magnification (Figures 1d–h). A Raman image constructed using the 2848 cm^–1^ band intensity is shown in Figure 1f. Interestingly, a high 2848 cm^–1^ signal was observed at the location of amyloid plaques, which might be surprising since the 2848 cm^–1^ Raman band is not typically associated with proteins but originates from the symmetric CH_2_ stretching of lipids (see Table S1 for the assignment of the Raman bands). However, it has been previously shown that amyloid plaques are often surrounded or colocalized with a lipid-rich region.^14,16,17,29^Figure 1f also shows that the paraffin wax used to embed mouse brain generates the 2848 cm^–1^ Raman band (top-right corner). However, it is easy to distinguish mouse brain from the paraffin area in the optical image (Figure 1a,c).

We discovered that another effective method to identify amyloid plaques over large areas of the arcAβ mouse brain tissue is to use negative contrast of the Raman signals of the glass substrate. In the confocal Raman spectrum of the glass substrate shown in Figure S1, two prominent bands are present at 1097 and 557 cm^–1^, which are assigned to the symmetric stretching of silica tetrahedra and a combination of the stretching and bending of Si–O–Si bridging bonds, respectively.^30^ Raman images of the mouse brain slice constructed using the intensities of 1097 and 557 cm^–1^ bands are shown in Figure 1g,h, respectively. Notably, at the location of amyloid plaques, intensity of both borosilicate glass bands decreases presumably due to the relatively higher optical density of the amyloid plaques compared to the surrounding brain tissue. According to the diffraction theory, developed by Abbe^31^ and Rayleigh,^32,33^ axial resolution of a confocal optical microscope is defined as 2λ/NA,^2^ where λ is the wavelength of excitation laser and NA is the numerical aperture of objective lens.^34^ For these measurements, we used a 532 nm excitation laser and a 5×, 0.15 NA objective lens, which provided an axial resolution of 47.3 μm. Given that the brain slice thickness is only 1.5 μm, it is not surprising that the Raman signals of the glass substrate are detected in Figure 1g,h. These results demonstrate that Raman imaging can directly identify amyloid plaques over areas of several hundred μm in the brain tissue without the need for staining or any complementary technique like μFTIR microscopy as reported previously.^14^

**Figure 2:**
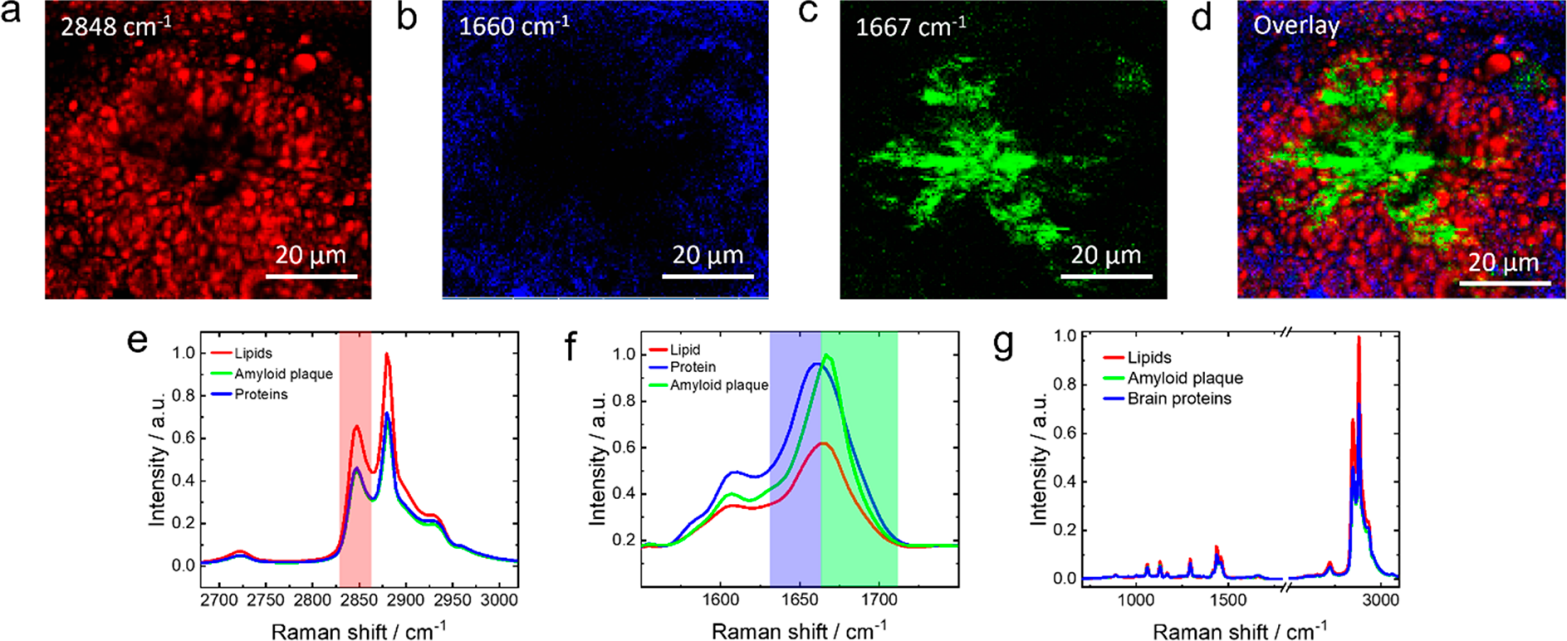
Confocal Raman images of the arcAβ mouse brain slice constructed using the intensities of the Raman bands at (a) 2848, (b) 1660, and (c) 1667 cm^–1^. (d) Overlay of the confocal Raman images shown in panels a–c. Laser power: 19 mW. Acquisition time: 1 s. Step size: 500 nm. (e) The C–H stretching region of the average Raman spectra of the areas populated with (red trace) lipids, (green trace) plaques, and (blue trace) proteins. The Raman band at 2848 cm^–1^, used to construct the image shown in panel a, is highlighted with red stripe. (f) The amide I spectral region of the average Raman spectra of the sample areas populated with (red trace) lipids, (green trace) amyloid plaques, and (blue trace) proteins. The Raman bands at 1660 and 1667 cm^–1^ used to construct the images shown in panels b and c, respectively, are highlighted with blue and green stripes. (g) Average Raman spectra of the areas populated with lipids (red trace), amyloid plaques (green trace), and proteins (blue trace) showing both fingerprint and C–H stretching regions.

Although the amyloid plaques can be identified over sample areas of several hundred μm using confocal Raman imaging with the 5× magnification objective lens, the spectral signal to noise (S/N) ratio remains quite low, thus providing only limited structural information. For a more detailed investigation of the amyloid plaques, confocal Raman imaging was performed using an objective lens with 100× magnification, which provided a significantly higher S/N ratio in the measured spectra. Higher resolution confocal Raman images of an amyloid plaque region in the arcAβ mouse brain constructed using intensities of the Raman bands at 2848, 1660, and 1667 cm^–1^ are shown in Figure 2a–c, respectively. An overlay of these images is presented in Figure 2d. As discussed above, the signal at 2848 cm^–1^ is the marker band for lipids, which is highlighted in red in the Raman spectra plotted in Figure 2e. The other two signals at 1660 and 1667 cm^–1^ belonging to the amide I region, characteristic of proteins, are highlighted in Figure 2f. Average Raman spectra showing both the fingerprint and the C–H regions are displayed in Figure 2g. For the ease of of discussion, we will refer to all non-amyloid proteins as proteins. It has been previously shown that, in amyloid plaques, the amide I band is shifted toward higher wavenumbers compared to other proteins.^17^ We indeed observe a blue-shift of 7 cm^–1^ at the location of amyloid plaques as shown in the average Raman spectra fitted using Lorentzian curves in Figure S2. Based on this, we denote the Raman bands at 1660 and 1667 cm^–1^ as the markers for proteins and amyloid plaques, respectively. Interestingly, a clear spatial segregation of the lipid, protein, and amyloid plaque regions is observed in Figure 2a–c. The overlay image in Figure 2d shows it even more distinctly. The amyloid plaque (green) is surrounded by a lipid-rich region (red), which in turn is encapsulated by a protein-rich region (blue). Notably, the 1660 cm^–1^ band can arise either from the C=O stretching of proteins or C=C stretching of lipids. However, the spatial distribution of the 1660 cm^–1^ signal (Figure 2b) is clearly distinct from that of the 2850 cm^–1^ signal (Figure 2a, unique for lipids). Therefore, we can reasonably assign the 1660 cm^–1^ signal to the amide I band of proteins, which was not clear in the previous reports.^17^ The optical images before and after confocal Raman imaging showed no changes in the sample features, confirming the absence of any photodamage (Figure S3). Furthermore, no sign of sample heating or photodegradation was observed in the measured spectra, which typically manifests as shifting and/or broadening of Raman peaks or an increase in the spectral background over time. The presence of a lipid halo around amyloid plaques (Figure 2d) has also been reported before.^14,17^ However, the lipid content within and around the amyloid plaques has been found to vary considerably. For example, no increase in the lipid content either in or around amyloid plaques was observed in some studies,^12,26^ whereas in another study, lipid aggregates were found to be colocalized with the amyloid plaques.^16^

**Figure 3:**
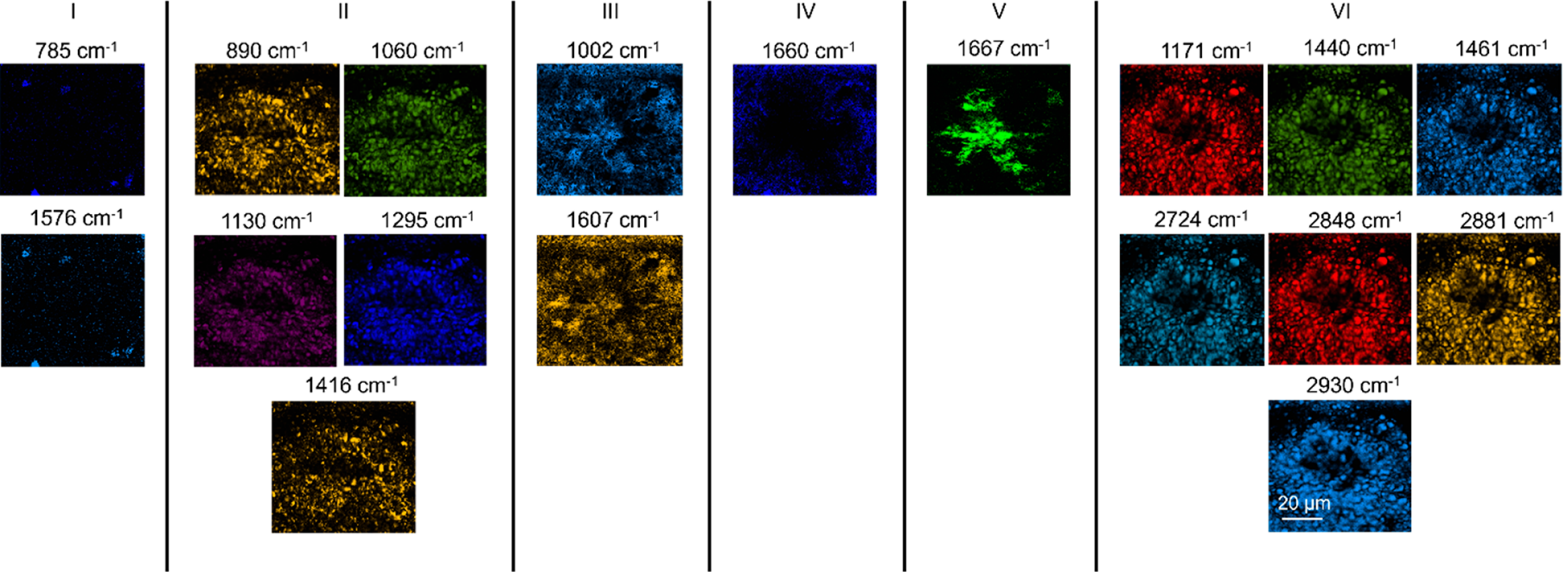
Confocal Raman images of the arcAβ mouse brain slice constructed using the intensities of all bands detected in the spectra. Laser power: 19 mW. Acquisition time: 1 s. Step size: 500 nm. Based on spatial correlation of Raman signals, the images are classified into 6 different groups. The group I signals originate from nucleic acids. The group II signals originate from lipids. The group III signals originate from proteins. The group IV signal originates from proteins. The group V signal originates from amyloid plaques. The group VI signals originate from lipids.

Figure 2g displays average Raman spectra (both fingerprint and C–H stretching regions) of the lipid-, amyloid plaque-, and protein-rich areas shown in Figure 2a–c. Raman images constructed using intensities of all bands detected in the spectra are presented in Figure 3. Raman images were classified into 6 groups based on the spatial correlation of Raman signals. Group I consists of images constructed using the bands at 785 and 1576 cm^–1^, which represent the vibrational modes of the nucleic acids. In this case, the analyte identification is straightforward. Similarly, in groups IV and V, proteins are clearly distinguished from amyloid plaques, as discussed earlier. Group III consists of Raman images constructed using the bands at 1002 and 1607 cm^–1^, which are protein signals that originate from tyrosine (Tyr), tryptophan (Trp), or phenylalanine (Phe) amino acids. The comparison of Raman images in groups III, IV, and V indicates that the signals of group III are detected in both proteins and amyloid plaque areas and, therefore, cannot be unambiguously used as Raman markers for any of them. In groups II and VI, Raman signals at 1060, 1295, 1416, 1440, 2724, and 2848 cm^–1^ originate from lipids, whereas the signals at 890, 1130, 1171, 1461, 2881, and 2930 cm^–1^ can originate from either lipids or proteins. However, the latter group of signals is classified in group II and VI because their spatial distribution in the Raman images correlates very well with the purely lipid signals. Interestingly, there is no perfect spatial match between the signals in groups II and VI even though they all represent lipid vibrations. The reason for this spatial mismatch is not entirely clear but could possibly arise from different types of lipids or structurally different lipids, i.e., lipids with different conformation or packing. To check reproducibility, we performed hyperspectral Raman imaging in another sample region, which also showed spatial segregation of the lipids, proteins, and amyloid plaques. The corresponding data and analysis are presented in Figures S4 and S5.

**Figure 4:**
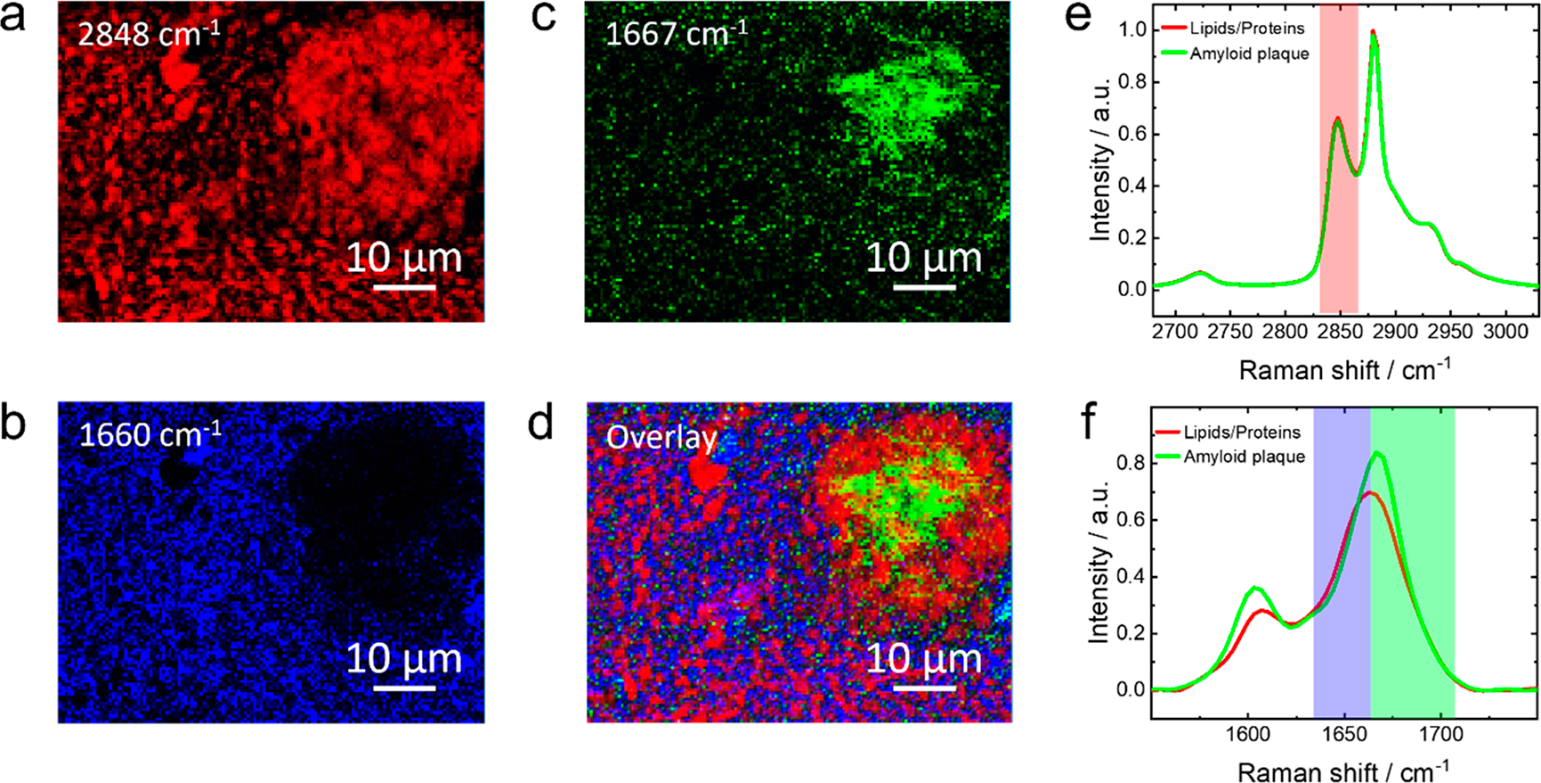
Confocal Raman images of a different region of the arcAβ mouse brain slice constructed using the intensities of Raman bands at (a) 2848, (b) 1660, and (c) 1667 cm^–1^. (d) Overlay of the confocal Raman images shown in panels a–c. Laser power: 19 mW. Acquisition time: 1 s. Step size: 500 nm. (e) C–H stretching region of the average Raman spectra of the areas populated with lipids/proteins (red trace) and amyloid plaque (green trace). The Raman band at 2848 cm^–1^ used to construct the image shown in panel a is highlighted with red stripe. (f) The amide I spectral region of the average Raman spectra of the sample areas populated with lipids/proteins (red trace) and amyloid plaque (green trace). The Raman bands at 1660 and 1667 cm^–1^ used to construct the images shown in panels b and c, respectively are highlighted with blue and green stripes.

The data presented in Figure 4 illustrate hyperspectral Raman imaging of an amyloid plaque in a different region of the mouse brain slice. The confocal Raman images depicted in Figure 4a–c were generated by using Raman markers for proteins, lipids, and amyloid plaques at 1660, 2848, and 1667 cm^–1^, respectively. Figure 4d provides an overlay of the three Raman images and the Raman bands for proteins, lipids, and amyloid plaques are labeled in Figure 4e,f. As demonstrated in Figure 4c,d, the proteins and amyloid plaque are clearly distinguished in this region of the arcAβ mouse brain, just like the amyloid plaque region in Figure 2a–d. However, no discernible spatial separation between the lipids and the amyloid plaque is observed in Figure 4a and c, where the amyloid plaque entirely overlaps the lipid-rich area. Figures S6 and S7 demonstrate a similar distribution of proteins, lipids, and amyloid plaques in another amyloid plaque region. These findings illustrate that amyloid plaques in the arcAβ mouse brain can have significantly different chemical compositions.

**Figure 5:**
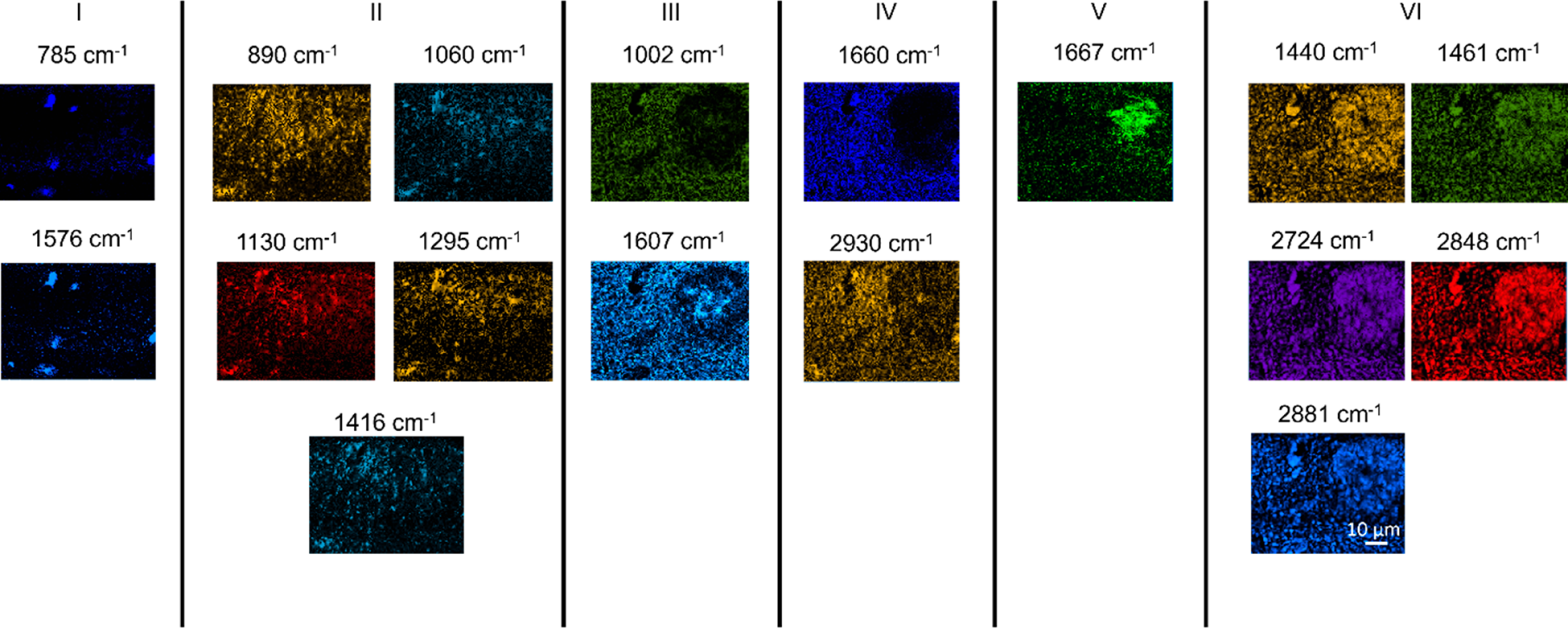
Confocal Raman images of the arcAβ mouse brain slice constructed using the intensities of all Raman bands measured in the amyloid plaque region shown in Figure 4. Laser power: 19 mW. Acquisition time: 1 s. Step size: 500 nm. Based on the spatial correlation between Raman signals, the images are classified into 6 groups. The group I signals originate from nucleic acids. The group II signals originate from lipids. The group III signals originate from proteins. The group IV signal originate from proteins. The group V signal originates from amyloid plaques. The group VI signals originate from lipids.

Like Figure 3, the Raman signals measured in the region of the amyloid plaque presented in Figure 4 were segregated into six groups based on the spatial correlation of the Raman images as shown in Figure 5. Nonetheless, there are some notable differences between the trends exhibited in Figures 3 and 5. For instance, the group III signals differ between the sample areas in Figures 3 and 5. In Figure 3, the 1002 and 1607 cm^–1^ signals displayed comparable intensity in the areas rich in proteins and amyloid plaques. However, in Figure 5, only the 1607 cm^–1^ signal exhibited a similar intensity in the protein and amyloid plaque rich areas, while the 1002 cm^–1^ signal (Phe marker) was considerably lower in amyloid plaque rich area. Given that amyloid plaques are characterized by a distinctive structure consisting of β-sheet-rich fibrils,^35,36^ and their primary structure is not expected to differ significantly in different plaques, the observed differences in the Phe content in Figures 3 and 5 imply different concentrations of native brain proteins in the amyloid plaque rich areas. Indeed, previously different extracellular proteins have been found to colocalize with amyloid plaques.^37^ Therefore, it is possible that Phe-containing protein was colocalized with the amyloid plaque in Figure 3, which was not the case for the plaque in Figure 5. Further analysis in the brain tissue from a sporadic and autosomal dominant AD patient, different stages of AD, and different strains of amyloidosis mouse models of AD will provide additional insights.^38,39^

Another difference between the sample regions in Figures 3 and 5 is identified in the group VI signals. Unlike Figure 3, the 2930 cm^–1^ signal is not spatially congruent with the other lipid signals from group VI in Figure 5. Instead, it correlates with the protein signal at 1660 cm^–1^ from group IV. The 2930 cm^–1^ signal arises from CH_3_ symmetric stretching (Table S1), which could be present in both lipids and proteins. It is unclear why the 2930 cm^–1^ signal spatially matches lipid signals in some brain regions (Figure 3) while it matches protein signals in the other regions (Figure 5). Nonetheless, it is certain that the 2930 cm^–1^ band is not a reliable Raman marker for either proteins or lipids in samples containing both.

## 3 Conclusions

In this study, we have demonstrated that hyperspectral Raman imaging is a cost-effective, label-free, and noninvasive tool for the chemical characterization of amyloid plaques in the brain tissue of the arcAβ mouse model of AD. Our results indicate that Raman imaging is highly proficient in identifying amyloid plaques over large sample areas without the need for a supplementary technique such as μFTIR. Moreover, by utilizing the spatial correlation between Raman signals, we were able to unequivocally distinguish proteins, lipids, and amyloid plaques within the complex chemical environment of the transgenic mouse brain tissue. Noteworthy, we chose not to use principal component analysis (PCA) to avoid overcomplicating spectral analysis since PCA is known to suffer from numerous limitations in the context of Raman data including negative values, inverse peaks, physically unrealistic results, sensitivity to outliers, lack of clear physical/chemical interpretation of the principal components, loss of spatial information particularly for heterogeneous biological materials, etc.^40,41^ However, our easy and straightforward analytical methodology can reveal not only variations in the shape and size of amyloid plaques, as previously reported, but also differences in their chemical composition. To the best of our knowledge, this is the first demonstration of successfully discriminating different types of amyloid plaques based on their lipid and protein content. We believe that this work will accelerate the application of confocal Raman imaging in the chemical characterization and analysis of protein misfolding pathologies.

## 4 Materials and Methods

### 4.1 Animal Model

Brain of a transgenic arcAβ mouse overexpressing the human APP695 transgene containing the Arctic (E693G) mutation under the control of prion protein was investigated in this study. Animals were housed in an individual ventilated cage inside a temperature-controlled room under a 12 h dark/light cycle with ad libitum access to food and water. All experiments were performed in accordance with the Swiss Federal Act on Animal Protection and approved by the Cantonal Veterinary Office Zurich (permit number: ZH162/20). Mouse was perfused under ketamine/xylazine (75/10/2 mg/kg or 50/4.5 mg/kg body weight, ip bolus injection) with ice-cold 0.1 M phosphate-buffered saline (PBS, pH 7.4) and 4% paraformaldehyde (PFA) in 0.1 M PBS (pH 7.4). After perfusion, the mouse was decapitated, and the brain was quickly removed and fixed for 1 day in 4% PFA (pH 7.4). Brain was then embedded in paraffin until sectioning as described earlier.^28^

### 4.2 Sample Preparation

Paraffin-embedded mouse brains were cut sagittally into 1.5 μm thick slices using an electronic motorized rotation microtome (HM 355S, Microm AG, Germany). Mouse brain slices were placed on borosilicate glass coverslips (thickness no. 1.5) for microanalysis using hyperspectral Raman imaging.

### 4.3 Immunofluorescence Staining and Fluorescence Microscopy

Brain tissues were stained with anti-Aβ1–16 antibody 6E10 (BioLegend, 803001, 1:750) and Donkey-anti-Mouse Alexa488 (Jackson, 715-545-151, 1:500) and counter-stained using 4′,6-diamidino-2-phenylindole (DAPI) for nuclei (Sigma, D9542–10MG, 1 μg/mL). The brain sections were imaged at 20× magnification using an Axio Observer Z1 microscope (whole brain slide). The images were analyzed by using Qupath.

### 4.4 Hyperspectral Raman Imaging

A LabRam Soleil instrument (Horiba Scientific, France) was used to perform confocal Raman measurements. A linearly polarized excitation laser beam of 532 nm wavelength was focused onto the sample surface by either a low-magnification (5×, 0.15 NA, Nikon, Japan) or high-magnification (100×, 0.9 NA, Nikon, Japan) objective lens. The objective lens used for sample irradiation also collected the Raman scattered light and directed it to the spectrometer equipped with a CCD detector. All Raman measurements were performed with a 600 lines/mm grating and a 100 μm pinhole.

### 4.5 Data Analysis

The Raman data analysis was performed in LabSpec 6.7.1.10 (Horiba Scientific, France). The Raman spectra were smoothed and baseline-corrected by subtracting a polynomial background from the raw spectra. All confocal Raman images were constructed using baseline-subtracted peak height values.

## Supporting Information Available

Table of Raman peak assignments, reference Raman spectrum of borosilicate glass substrate, optical images of the sample before and after Raman imaging, and additional Raman imaging data sets (PDF)

## References

[ref1] Scheltens P.; Blennow K.; Breteler M. M. B.; de Strooper B.; Frisoni G. B.; Salloway S.; Van der Flier W. M. Alzheimer’s Disease. Lancet 2016, 388 (10043), 505–517. 10.1016/S0140-6736(15)01124-1. 26921134

[ref2] Patterson C. World Alzheimer Report 2018: The State of the Art of Dementia Research: New Frontiers; Alzheimer’s Disease International: London, 2018.

[ref3] Cummings J. L. Alzheimer’s Disease. N. Engl. J. Med. 2004, 351 (1), 56–67. 10.1056/NEJMra040223. 15229308

[ref4] Ikonomovic M. D.; Klunk W. E.; Abrahamson E. E.; Mathis C. A.; Price J. C.; Tsopelas N. D.; Lopresti B. J.; Ziolko S.; Bi W.; Paljug W. R.; Debnath M. L.; Hope C. E.; Isanski B. A.; Hamilton R. L.; DeKosky S. T. Post-Mortem Correlates of in Vivo PiB-PET Amyloid Imaging in a Typical Case of Alzheimer’s Disease. Brain 2008, 131 (6), 1630–1645. 10.1093/brain/awn016. 18339640 PMC2408940

[ref5] Hanseeuw B. J.; Betensky R. A.; Jacobs H. I. L.; Schultz A. P.; Sepulcre J.; Becker J. A.; Cosio D. M. O.; Farrell M.; Quiroz Y. T.; Mormino E. C.; Buckley R. F.; Papp K. V.; Amariglio R. A.; Dewachter I.; Ivanoiu A.; Huijbers W.; Hedden T.; Marshall G. A.; Chhatwal J. P.; Rentz D. M.; Sperling R. A.; Johnson K. Association of Amyloid and Tau with Cognition in Preclinical Alzheimer Disease: A Longitudinal Study. JAMA Neurol. 2019, 76 (8), 915–924. 10.1001/jamaneurol.2019.1424. 31157827 PMC6547132

[ref6] Nabers A.; Hafermann H.; Wiltfang J.; Gerwert K. Aβ and Tau Structure-Based Biomarkers for a Blood- and CSF-Based Two-Step Recruitment Strategy to Identify Patients with Dementia Due to Alzheimer’s Disease. Alzheimer’s Dement. Diagnosis, Assess. Dis. Monit. 2019, 11, 257–263. 10.1016/j.dadm.2019.01.008. PMC6416642 30911600

[ref7] Mecocci P.; Paolacci L.; Boccardi V. Biomarkers of Dementia: From Bench to Clinical Side. Geriatr. Care 2018, 4 (2), 1 10.4081/gc.2018.7718.

[ref8] Mordechai S.; Shufan E.; Porat Katz B. S.; Salman A. Early Diagnosis of Alzheimer’s Disease Using Infrared Spectroscopy of Isolated Blood Samples Followed by Multivariate Analyses. Analyst 2017, 142 (8), 1276–1284. 10.1039/C6AN01580H. 27827489

[ref9] Ryzhikova E.; Kazakov O.; Halamkova L.; Celmins D.; Malone P.; Molho E.; Zimmerman E. A.; Lednev I. K. Raman Spectroscopy of Blood Serum for Alzheimer’s Disease Diagnostics: Specificity Relative to Other Types of Dementia. J. Biophotonics 2015, 8 (7), 584–596. 10.1002/jbio.201400060. 25256347 PMC4575592

[ref10] den Haan J.; Morrema T. H. J.; Rozemuller A. J.; Bouwman F. H.; Hoozemans J. J. M. Different Curcumin Forms Selectively Bind Fibrillar Amyloid Beta in Post Mortem Alzheimer’s Disease Brains: Implications for in-Vivo Diagnostics. Acta Neuropathol. Commun. 2018, 6 (1), 75 10.1186/s40478-018-0577-2. 30092839 PMC6083624

[ref11] Alafuzoff I.; Thal D. R.; Arzberger T.; Bogdanovic N.; Al-Sarraj S.; Bodi I.; Boluda S.; Bugiani O.; Duyckaerts C.; Gelpi E.; Gentleman S.; Giaccone G.; Graeber M.; Hortobagyi T.; Höftberger R.; Ince P.; Ironside J. W.; Kavantzas N.; King A.; Korkolopoulou P.; Kovács G. G.; Meyronet D.; Monoranu C.; Nilsson T.; Parchi P.; Patsouris E.; Pikkarainen M.; Revesz T.; Rozemuller A.; Seilhean D.; Schulz-Schaeffer W.; Streichenberger N.; Wharton S. B.; Kretzschmar H. Assessment of β-Amyloid Deposits in Human Brain: A Study of the BrainNet Europe Consortium. Acta Neuropathol. 2009, 117 (3), 309–320. 10.1007/s00401-009-0485-4. 19184666 PMC2910889

[ref12] Lochocki B.; Boon B. D. C.; Verheul S. R.; Zada L.; Hoozemans J. J. M.; Ariese F.; de Boer J. F. Multimodal, Label-Free Fluorescence and Raman Imaging of Amyloid Deposits in Snap-Frozen Alzheimer’s Disease Human Brain Tissue. Commun. Biol. 2021, 4 (1), 1–13. 10.1038/s42003-021-01981-x. 33859370 PMC8050064

[ref13] Zhang X.; Zeng F.; Li Y.; Qiao Y. Improvement in Focusing Accuracy of DNA Sequencing Microscope with Multi-Position Laser Differential Confocal Autofocus Method. Opt. Express 2018, 26 (2), 887 10.1364/OE.26.000887. 29401968

[ref14] Palombo F.; Tamagnini F.; Jeynes J. C. G.; Mattana S.; Swift I.; Nallala J.; Hancock J.; Brown J. T.; Randall A. D.; Stone N. Detection of Aβ Plaque-Associated Astrogliosis in Alzheimer’s Disease Brain by Spectroscopic Imaging and Immunohistochemistry. Analyst 2018, 143 (4), 850–857. 10.1039/C7AN01747B. 29230441 PMC5851084

[ref15] Kato R.; Yano T.; Minamikawa T.; Tanaka T. High-Sensitivity Hyperspectral Vibrational Imaging of Heart Tissues by Mid-Infrared Photothermal Microscopy. Anal. Sci. 2022, 38 (12), 1497–1503. 10.1007/s44211-022-00182-8. 36070070

[ref16] Kiskis J.; Fink H.; Nyberg L.; Thyr J.; Li J. Y.; Enejder A. Plaque-Associated Lipids in Alzheimer’s Diseased Brain Tissue Visualized by Nonlinear Microscopy. Sci. Rep. 2015, 5, 1–9. 10.1038/srep13489. PMC4550829 26311128

[ref17] Ji M.; Arbel M.; Zhang L.; Freudiger C. W.; Hou S. S.; Lin D.; Yang X.; Bacskai B. J.; Xie X. S. Label-Free Imaging of Amyloid Plaques in Alzheimer’s Disease with Stimulated Raman Scattering Microscopy. Sci. Adv. 2018, 4 (11), 1–9. 10.1126/sciadv.aat7715. PMC6239428 30456301

[ref18] Kumar N.; Drozdz M. M.; Jiang H.; Santos D. M.; Vaux D. J. Nanoscale Mapping of Newly-Synthesised Phospholipid Molecules in a Biological Cell Using Tip-Enhanced Raman Spectroscopy. Chem. Commun. 2017, 53 (16), 2451–2454. 10.1039/C6CC10226C. 28177338

[ref19] Mrđenović D.; Tang Z.-X.; Pandey Y.; Su W.; Zhang Y.; Kumar N.; Zenobi R. Regioselective Tip-Enhanced Raman Spectroscopy of Lipid Membranes with Sub-Nanometer Axial Resolution. Nano Lett. 2023, 23 (9), 3939–3946. 10.1021/acs.nanolett.3c00689. 37096805

[ref20] Pandey Y.; Kumar N.; Goubert G.; Zenobi R. Nanoscale Chemical Imaging of Supported Lipid Monolayers Using Tip-Enhanced Raman Spectroscopy. Angew. Chemie Int. Ed. 2021, 60 (35), 19041–19046. 10.1002/anie.202106128. PMC8456802 34170590

[ref21] Kato R.; Moriyama T.; Umakoshi T.; Yano T.; Verma P. Ultrastable Tip-Enhanced Hyperspectral Optical Nanoimaging for Defect Analysis of Large-Sized WS2 Layers. Sci. Adv. 2022, 8 (28), eabo4021 10.1126/sciadv.abo4021. 35857514 PMC9286508

[ref22] Mrđenović D.; Ge W.; Kumar N.; Zenobi R. Nanoscale Chemical Imaging of Human Cell Membranes Using Tip-Enhanced Raman Spectroscopy. Angew. Chemie Int. Ed. 2022, 61 (43), e202210288 10.1002/anie.202210288. PMC9826433 36057139

[ref23] Tu H.; Boppart S. A. Coherent Anti-Stokes Raman Scattering Microscopy: Overcoming Technical Barriers for Clinical Translation. J. Biophotonics 2014, 7 (1–2), 9–22. 10.1002/jbio.201300031. 23674234 PMC4486077

[ref24] Hosokawa M.; Ando M.; Mukai S.; Osada K.; Yoshino T.; Hamaguchi H. O.; Tanaka T. In Vivo Live Cell Imaging for the Quantitative Monitoring of Lipids by Using Raman Microspectroscopy. Anal. Chem. 2014, 86 (16), 8224–8230. 10.1021/ac501591d. 25073083

[ref25] Wu H.; Volponi J. V.; Oliver A. E.; Parikh A. N.; Simmons B. A.; Singh S. In Vivo Lipidomics Using Single-Cell Raman Spectroscopy. Proc. Natl. Acad. Sci. U. S. A. 2011, 108 (9), 3809–3814. 10.1073/pnas.1009043108. 21310969 PMC3048102

[ref26] Michael R.; Lenferink A.; Vrensen G. F. J. M.; Gelpi E.; Barraquer R. I.; Otto C. Hyperspectral Raman Imaging of Neuritic Plaques and Neurofibrillary Tangles in Brain Tissue from Alzheimer’s Disease Patients. Sci. Rep. 2017, 7 (1), 15603 10.1038/s41598-017-16002-3. 29142266 PMC5688091

[ref27] Lu F. K.; Basu S.; Igras V.; Hoang M. P.; Ji M.; Fu D.; Holtom G. R.; Neel V. A.; Freudiger C. W.; Fisher D. E.; Xie X. S. Label-Free DNA Imaging in Vivo with Stimulated Raman Scattering Microscopy. Proc. Natl. Acad. Sci. U. S. A. 2015, 112 (43), 11624–11629. 10.1073/pnas.1515121112. 26324899 PMC4577158

[ref28] Ni R.; Chen Z.; Deán-Ben X. L.; Voigt F. F.; Kirschenbaum D.; Shi G.; Villois A.; Zhou Q.; Crimi A.; Arosio P.; Nitsch R. M.; Nilsson K. P. R.; Aguzzi A.; Helmchen F.; Klohs J.; Razansky D. Multiscale Optical and Optoacoustic Imaging of Amyloid-β Deposits in Mice. Nat. Biomed. Eng. 2022, 6 (9), 1031–1044. 10.1038/s41551-022-00906-1. 35835994

[ref29] Schweikhard V.; Baral A.; Krishnamachari V.; Hay W. C.; Fuhrmann M.; Ji M.; Arbel M.; Zhang L.; Freudiger C. W.; Hou S. S.; Lin D.; Yang X.; Bacskai B. J.; Xie X. S.; Schweikhard V.; Baral A.; Krishnamachari V.; Hay W. C.; Fuhrmann M. Label-Free Characterization of Amyloid-β-Plaques and Associated Lipids in Brain Tissues Using Stimulated Raman Scattering Microscopy. bioRxiv 2019, 4 (11), eaat7715 10.1101/789248.

[ref30] White W. B.; Minser D. G. Raman Spectra and Structure of Natural Glasses. J. Non. Cryst. Solids 1984, 67 (1–3), 45–59. 10.1016/0022-3093(84)90140-6.

[ref31] Abbe E. Beiträge Zur Theorie Des Mikroskops Und Der Mikroskopischen Wahrnehmung: I. Die Construction von Mikroskopen Auf Grund Der Theorie. Arch. für mikroskopische Anat. 1873, 9 (1), 413–418. 10.1007/BF02956173.

[ref32] Rayleigh L. XXXI. Investigations in Optics, with Special Reference to the Spectroscope. London, Edinburgh, Dublin Philos. Mag. J. Sci. 1879, 8 (49), 261–274. 10.1080/14786447908639684.

[ref33] Rayleigh L. XV. On the Theory of Optical Images, with Special Reference to the Microscope. London, Edinburgh, Dublin Philos. Mag. J. Sci. 1896, 42 (255), 167–195. 10.1080/14786449608620902.

[ref34] Bessmeltsev V. P.; Maksimov M. V.; Vileiko V. V.; Goloshevskii N. V.; Terent’ev V. S. Multichannel Confocal Microscope Based on a Diffraction Focusing Multiplier with Automatic Synchronization of Scanning. Optoelectron. Instrum. Data Process. 2018, 54 (6), 531–537. 10.3103/S8756699018060018.

[ref35] Colvin M. T.; Silvers R.; Ni Q. Z.; Can T. V.; Sergeyev I.; Rosay M.; Donovan K. J.; Michael B.; Wall J.; Linse S.; Griffin R. G. Atomic Resolution Structure of Monomorphic Aβ 42 Amyloid Fibrils. J. Am. Chem. Soc. 2016, 138 (30), 9663–9674. 10.1021/jacs.6b05129. 27355699 PMC5389415

[ref36] Wälti M. A.; Ravotti F.; Arai H.; Glabe C. G.; Wall J. S.; Böckmann A.; Güntert P.; Meier B. H.; Riek R. Atomic-Resolution Structure of a Disease-Relevant Aβ(1–42) Amyloid Fibril. Proc. Natl. Acad. Sci. U. S. A. 2016, 113 (34), E4976-E4984 10.1073/pnas.1600749113. 27469165 PMC5003276

[ref37] Rahman M. M.; Lendel C. Extracellular Protein Components of Amyloid Plaques and Their Roles in Alzheimer’s Disease Pathology. Mol. Neurodegener. 2021, 16 (1), 59 10.1186/s13024-021-00465-0. 34454574 PMC8400902

[ref38] Ni R.; Gillberg P.; Bogdanovic N.; Viitanen M.; Myllykangas L.; Nennesmo I.; Långström B.; Nordberg A. Amyloid Tracers Binding Sites in Autosomal Dominant and Sporadic Alzheimer’s Disease. Alzheimer’s Dementia 2017, 13 (4), 419–430. 10.1016/j.jalz.2016.08.006. 27693181

[ref39] Thal D. R.; Rüb U.; Orantes M.; Braak H. Phases of Aβ-Deposition in the Human Brain and Its Relevance for the Development of AD. Neurology 2002, 58 (12), 1791–1800. 10.1212/WNL.58.12.1791. 12084879

[ref40] Blee A. L.; Day J. C. C.; Flewitt P. E. J.; Jeketo A.; Megson-Smith D. Non-negative Assisted Principal Component Analysis: A Novel Method of Data Analysis for Raman Spectroscopy. J. Raman Spectrosc. 2021, 52 (6), 1135–1147. 10.1002/jrs.6112.

[ref41] Prasad S.; Bruce L. M. Limitations of Principal Components Analysis for Hyperspectral Target Recognition. IEEE Geosci. Remote Sens. Lett. 2008, 5 (4), 625–629. 10.1109/LGRS.2008.2001282.

