# Supplementary material for: Probing Chemical Complexity of Amyloid Plaques in Alzheimer’s Disease Mice using Hyperspectral Raman Imaging
Source: ACS Chem Neurosci. 2023 Dec 14;15(1):78–85. doi: 10.1021/acschemneuro.3c00607 (PMC10767745; doi:10.1021/acschemneuro.3c00607)
Supplement: Supplementary file 1 — cn3c00607_si_001.pdf [file cn3c00607_si_001.pdf]

Supporting Information

Probing Chemical Complexity of Amyloid Plaques in Alzheimer's Disease Mice using Hyperspectral Raman Imaging

Dušan Mrđenović, [†] Benjamin F. Combes, [§] Ruiqing Ni, [§,#] Renato Zenobi, [†,*] and Naresh Kumar[†,*]

[†]Department of Chemistry and Applied Biosciences, ETH Zürich, Vladimir-Prelog-Weg 1–5/10, 8093 Zürich, Switzerland

[§]Institute for Regenerative Medicine, University of Zürich, Wagistrasse 12, 8952 Schlieren, Switzerland

[#]Institute for Biomedical Engineering, University of Zurich and ETH Zurich, Wolfgang-Pauli-Strasse 27, 8093 Zürich, Switzerland

Corresponding author e-mail address: zenobi@org.chem.ethz.ch, kumar@org.chem.ethz.ch

Table S1. Assignment of bands observed in the Raman spectra of mouse brain slices.[2–4]

Band position / cm^{-1}	Tentative assignment
785	Backbone O-P-O in nucleic acids
890	C-O-O skeletal (lipids), Trp (proteins)
1002	Ring breathing mode of Phe
1060	C-C stretch (lipids)
1130	C-C skeletal stretch (lipids), C-N stretch (proteins)
1171	C-C stretch (lipids), C-H in-plane bending mode of Tyr (protein)
1295	CH_2 twist (lipids)
1416	CH_2 bend (lipids)
1440	CH_2 bend (lipids)
1461	CH_2/CH_3 bend (lipids/proteins)
1576	Ring breathing of nucleic acids
1607	Tyr, Trp, Phe (proteins)
1660	Amide I (proteins), C=C stretch (lipids)
1667	Amide I of proteins containing β sheets
2724	C-H stretch (lipids)
2848	Symmetric CH_2 stretch (lipids)
2881	Asymmetric CH_2 stretch (lipids/proteins)
2930	Symmetric CH_3 stretch (lipids/proteins)

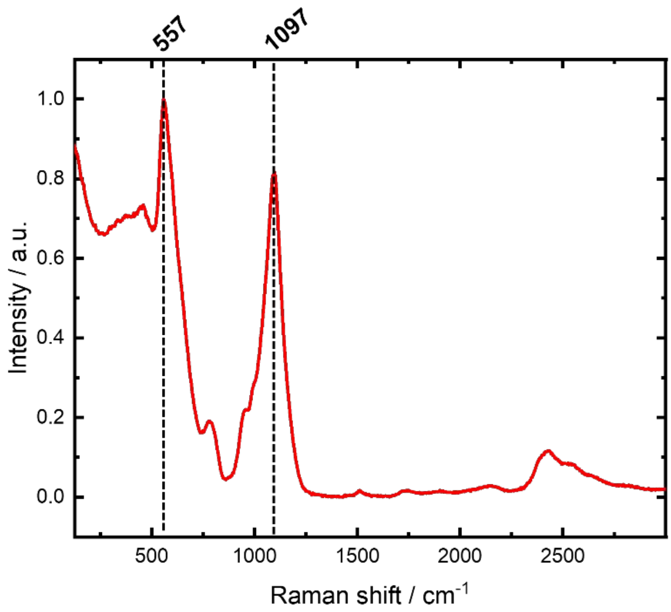

Figure S1. Confocal Raman spectrum of the borosilicate glass substrate used for supporting microtomed mouse brain slices in this work. Laser power: 77 mW. Acquisition time: 1 s.

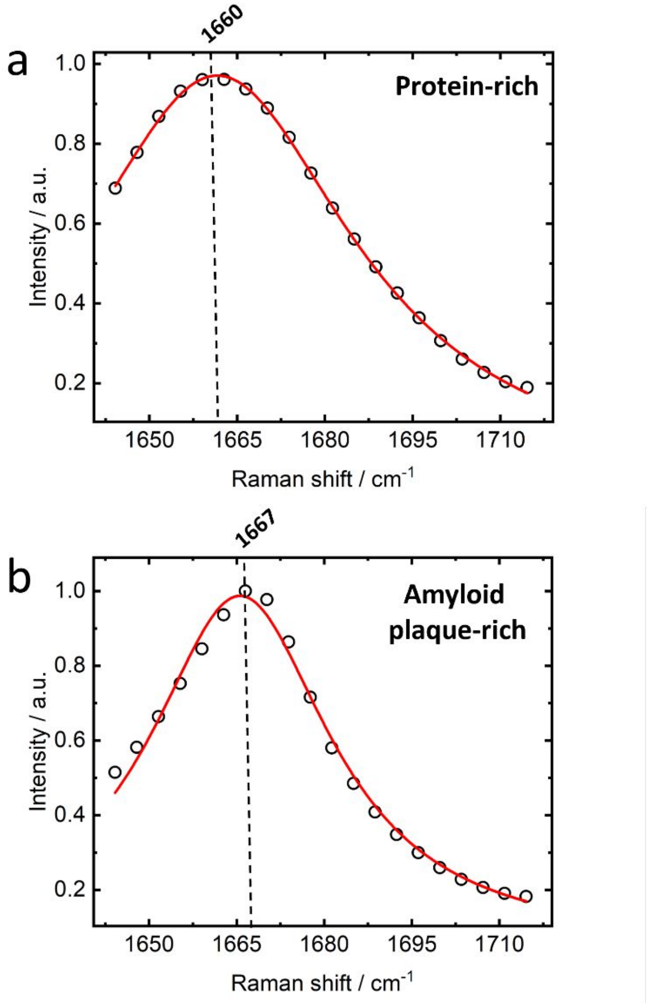

Figure S2. Average Raman spectra (1633 – 1713 cm^{-1}) measured in the protein-rich and amyloid plaque-rich regions shown in Figures 2b and 2c, respectively. Raman spectra are fitting using a Lorentzian function (red curve). A blue-shift of *ca.* 7 cm^{-1} is observed in position of Amide I band at the location of amyloid plaques.

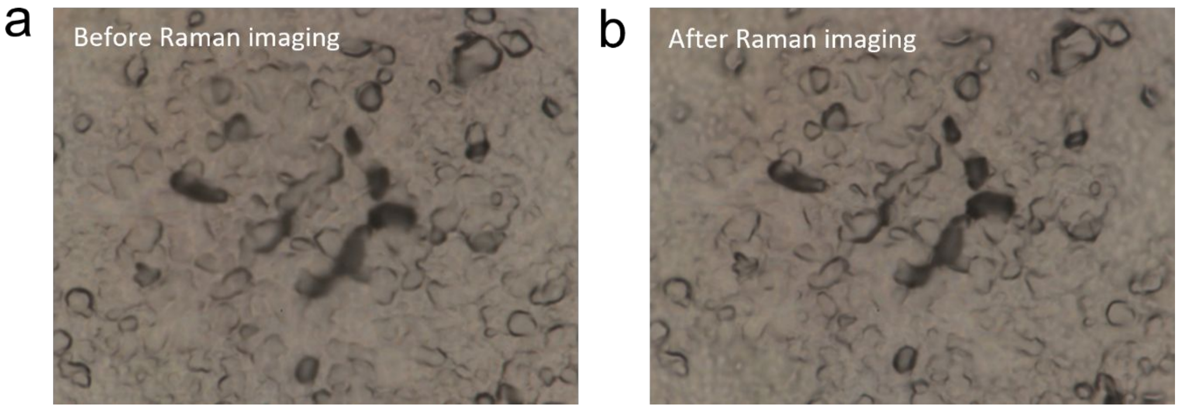

Figure S3. Optical images of the region presented in Figure 2a-d (a) before and (b) after confocal Raman imaging showing no sample damage.

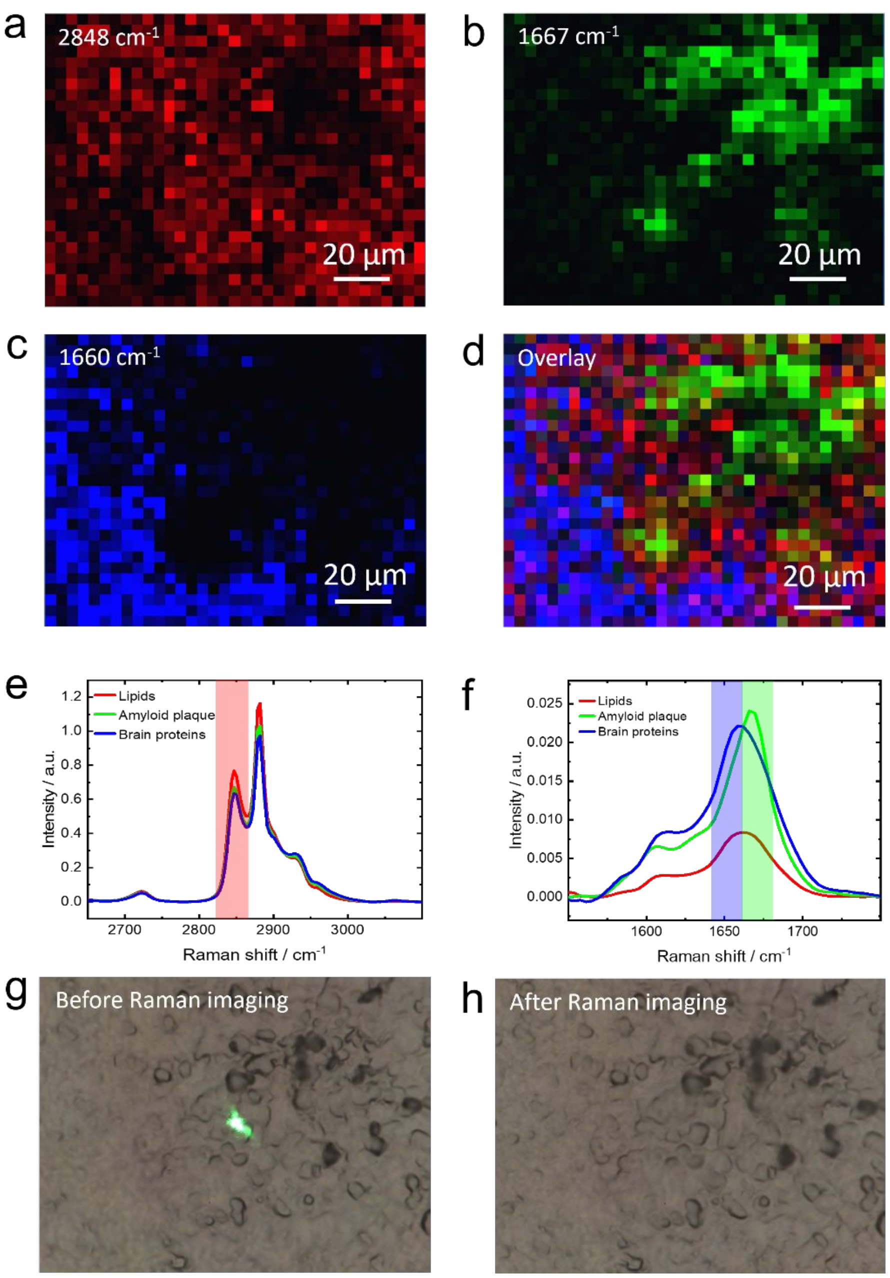

Figure S4. Confocal Raman images of lipid, amyloid plaque, and proteins in a different region of the arcAβ mouse brain slice constructed using the intensities of Raman bands at (a) 2848, (b) 1660, and (c) 1667 cm^{-1}, respectively. (d) Overlay of the confocal Raman images shown in Panels a-c. Laser power: 25 mW. Acquisition time: 1 s. Step size: 2 µm. Amyloid plaque (green) is surrounded by a lipid-rich region (red), which is further encapsulated by a protein-rich region (blue). (e) The C-H spectral region of the average Raman spectra of the areas populated with (red trace) lipids, (green trace) plaques, and (blue trace) proteins. The Raman band at 2848 cm^{-1}, used to construct the image shown in Panel a is highlighted in red. (f) The amide I spectral region of the average Raman spectra of the sample areas populated with (red trace) lipids, (green trace) amyloid plaques, and (blue trace) proteins. The Raman bands at 1660 and 1667 cm^{-1} used to construct the images shown in Panels b and c, respectively are highlighted in blue and green. Optical images of the measured region (g) before and (h) after confocal Raman imaging showing no sample damage. Focal spot of the green excitation laser is visible in Panel g.

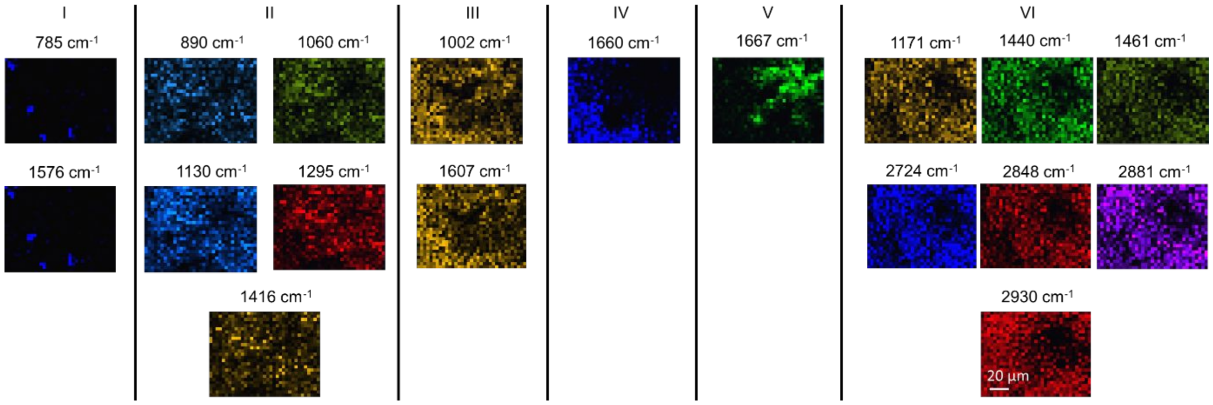

Figure S5. Confocal Raman images of the arcAβ mouse brain slice constructed using the intensities of all detected Raman bands in the region measured in Figure S4a-d. Laser power: 25 mW. Acquisition time: 1 s. Step size: 2 µm. Based on the spatial correlation between Raman signals, the images are classified into 6 groups. The group I signals originate from nucleic acids. The group II signals originate from lipids. The group III signals originate from proteins. The group IV signal originate from proteins. The group V signal originates from amyloid plaques. The group VI signals originate from lipids.

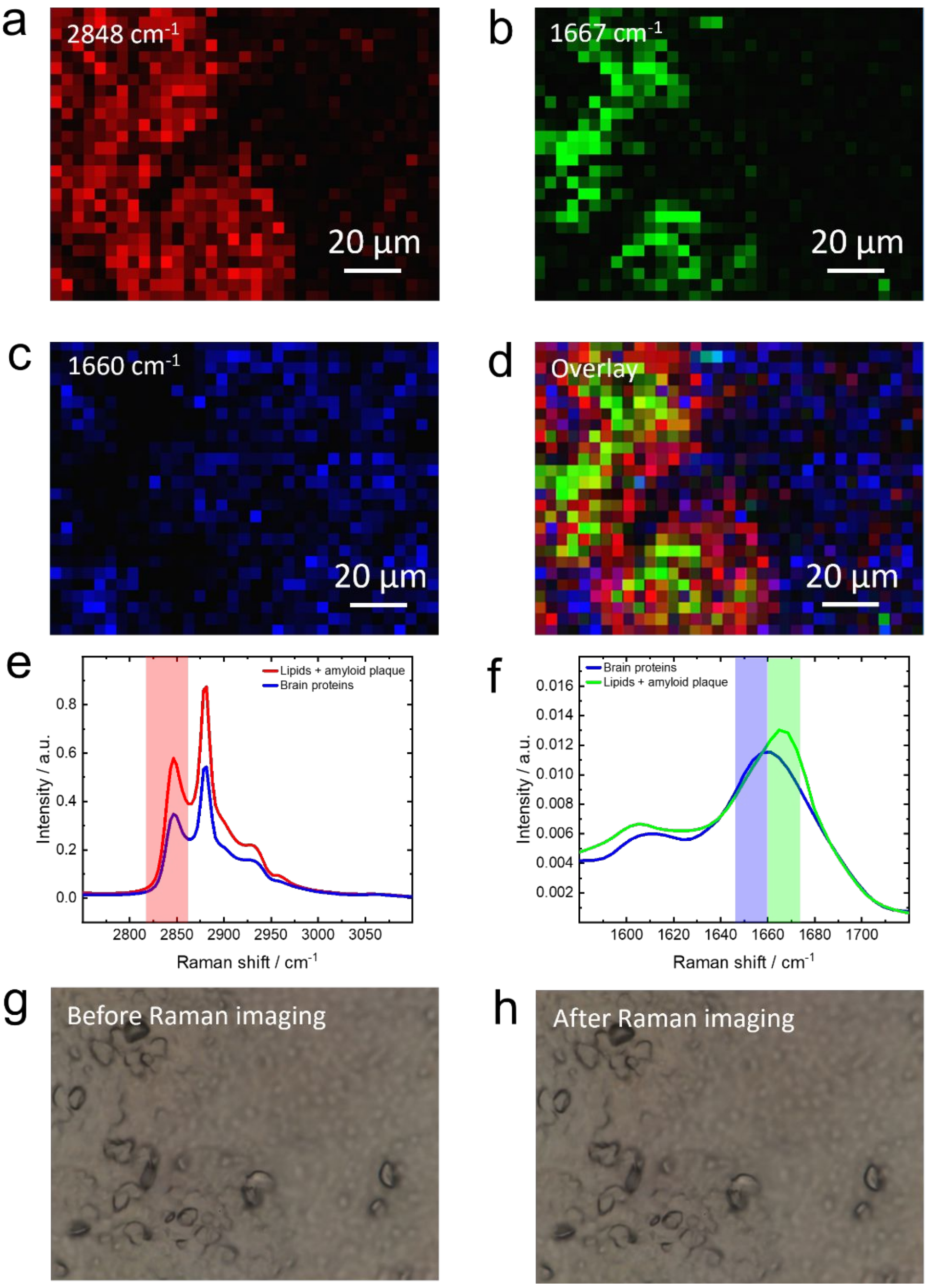

Figure S6. Confocal Raman images of lipid, amyloid plaque, and proteins in a different region of the arcAβ mouse brain slice constructed using the intensities of Raman bands at (a) 2848, (b) 1660, and (c) 1667 cm^{-1}, respectively. (d) Overlay of the confocal Raman images shown in Panels a-c. Laser power: 25 mW. Acquisition time: 1 s. Step size: 2 µm. Amyloid plaque (green) is surrounded by a lipid-rich region (red), which is further encapsulated by a protein-rich region (blue). (e) The C-H spectral region of the average Raman spectra of the areas populated with (red trace) lipids and amyloid plaque and (blue trace) proteins. The Raman band at 2848 cm^{-1}, used to construct the image shown in Panel a is highlighted in red. (f) The amide I spectral region of the average Raman spectra of the sample areas populated with (green trace) lipids and amyloid plaque and (blue trace) proteins. The Raman bands at 1660 and 1667 cm^{-1} used to construct the images shown in Panels b and c, respectively are highlighted in blue and green. Optical images of the measured region (g) before and (h) after confocal Raman imaging showing no sample damage.

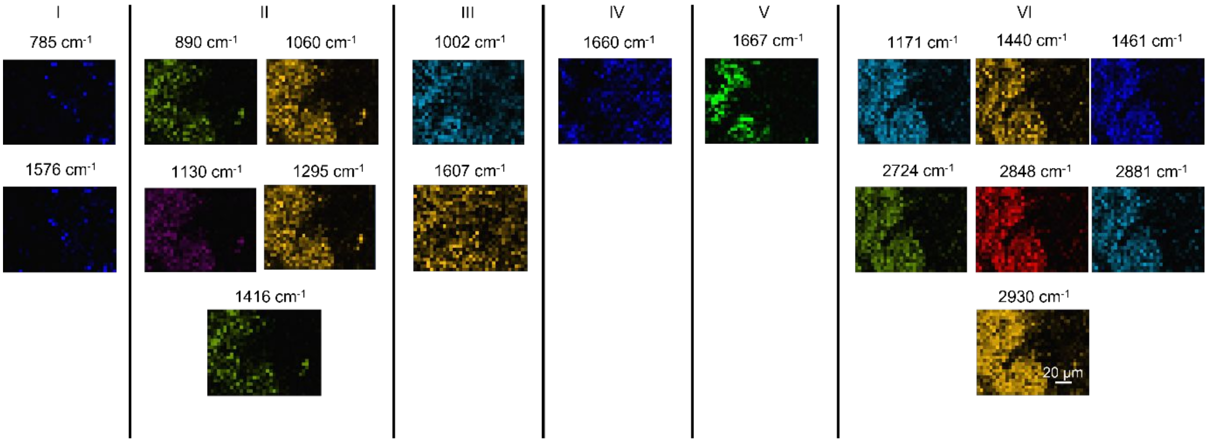

Figure S7. Confocal Raman images of the arcAβ mouse brain slice constructed using the intensities of all detected Raman bands in the region measured in Figure S6a-d. Laser power: 25 mW. Acquisition time: 1 s. Step size: 2 µm. Based on the spatial correlation between Raman signals, the images are classified into 6 groups. The group I signals originate from nucleic acids. The group II signals originate from lipids. The group III signals originate from proteins. The group IV signal originate from proteins. The group V signal originates from amyloid plaques. The group VI signals originate from lipids.

Supplementary references

[1] D. Mrđenović, W. Ge, N. Kumar, R. Zenobi, *Angew. Chemie Int. Ed.* **2022**, *61*, e202210288.

[2] K. Czamara, K. Majzner, M. Z. Pacia, K. Kochan, A. Kaczor, M. Baranska, *J. Raman Spectrosc.* **2015**, *46*, 4–20.

[3] A. Rygula, K. Majzner, K. M. Marzec, A. Kaczor, M. Pilarczyk, M. Baranska, *J. Raman Spectrosc.* **2013**, *44*, 1061–1076.

[4] Z. Movasaghi, S. Rehman, I. U. Rehman, *Appl. Spectrosc. Rev.* **2007**, *42*, 493–541.
